# Lambl’s Excrescences and Cardiac Papillary Fibroelastoma, an Unusual Cause of Embolic Strokes: Extended Data From a Single Center Case Series

**DOI:** 10.7759/cureus.89162

**Published:** 2025-07-31

**Authors:** Dhara Rana, Peter Pietrandrea, Rea Isaac, Ali Husain, Kristen Rainear

**Affiliations:** 1 Department of Internal Medicine, Inspira Medical Center Vineland, Vineland, USA; 2 Department of Cardiology, Deborah Heart and Lung Center, Browns Mills, USA; 3 School of Osteopathic Medicine, Rowan-Virtua School of Osteopathic Medicine, Glassboro, USA; 4 Department of Cardiology, Penn Cardiology, Vineland, USA

**Keywords:** cardiac papillary fibroelastomas (cpf), chronic kidney disease (ckd), coronary arterial disease (cad), diabetes mellitus (dm), hyperlipidemia (hld), hypertension (htn), lambl’s excrescences (le), transesophageal echocardiogram (tee)

## Abstract

Background

Lambl’s excrescences (LE) and cardiac papillary fibroelastomas (CPF) are valvular structures that have been associated with cryptogenic strokes, potentially acting as embolic sources or sites of thrombus formation. However, the management of LE and CPF - whether discovered incidentally or in the setting of cerebrovascular events (cardiovascular accident (CVA)/transient ischemic attack (TIA)) - remains poorly defined, with no randomized trials to guide treatment.

Methods

We conducted a retrospective case series at a single center from July 2018 to June 2024, identifying 10 patients with LE or CPF. Nine patients presented with CVA/TIA; one had an incidental CPF finding. All patients were followed for a minimum of six months. The cohort had a mean age of 69.4 years (range 58-79 years), and common comorbidities included hypertension (7/10), diabetes (5/10), hyperlipidemia (4/10), coronary artery disease (2/10), and chronic kidney disease (1/10).

Results

No alternative stroke etiology was identified in CVA/TIA cases after standard evaluation. Management strategies included direct oral anticoagulants (n=5), warfarin (n=1), dual antiplatelet therapy (n=3), and surgical valve replacement (n=1). No recurrent strokes related to LE or CPF were observed during follow-up. Two patients later experienced strokes due to progressive carotid artery stenosis.

Conclusions

In this small, descriptive case series, individualized management strategies for LE and CPF appeared safe over short- to mid-term follow-up. Given the absence of consensus guidelines and randomized data, further research is needed to define optimal treatment and clarify causal relationships.

## Introduction

Lambl’s excrescences (LE) and cardiac papillary fibroelastomas (CPF) are structures found on cardiac valves (most often the aortic and mitral valves) that have been implicated in cryptogenic strokes. These structures act as a nidus for clot formation or embolic structures themselves. If referred to cryptogenic stroke, prevalence jumps up to 40%, which has led many to suggest that valvular strands are the nidus for cryptogenic embolic strokes [1].

Most cases of LEs or CPFs are asymptomatic and are detected incidentally or in autopsy. Patients can present with stroke-like features such as motor deficits, sensory deficits, aphasia, dysarthria, dysphagia, visual disturbance, and cranial nerve palsies [1]. Cardioembolic stroke secondary to LEs or CPFs is a diagnosis of exclusion. When associated with stroke, an exhaustive stroke work-up to identify the potential cause of stroke should be taken, which can include carotid duplex ultrasound, a hypercoagulable workup, and a complete transesophageal echocardiogram (TEE) assessing the ascending, transverse, and arch segments of the aorta [1]. To diagnose a patient with LEs, a TEE is the gold standard. Alternatively, when TEE is not feasible, a high-resolution CT scan can be done. On TEE, LEs will appear as thin, hypermobile, filiform strands on the valvular cusps’ line of closure [1].

Various management strategies have been used, such as aspirin monotherapy, aspirin plus P2Y12 inhibitors, anticoagulation, or even valvular surgery. To date, no randomized clinical trial has been performed to evaluate each treatment option, and therefore, management is often left to the cardiologist’s discretion. The proposed case series aims to understand the presentation of LE and CPF and the treatment management.

## Materials and methods

This is a single-center descriptive study conducted by querying the institute's Electronic Medical Record (EMR) database to obtain cases with echocardiography showcasing LE or CPFs. The EMR was queried by keywords and International Classification of Diseases, 10th revision (ICD-10s) from 2018 to 2024 as seen in the appendix. The query revealed 35 unique records using ICD-10 codes (see Appendix). For each record, investigators looked for TTE that showed LE or CPF; if the query did not have them, the case was removed from the list. This left us with three patient records who had LE or CPF. In addition to the EMR query, we encountered seven cases from July 2019 to October 2023 in a hospital setting. A total of 10 unique cases were identified presenting with cardiovascular accident/transient ischemic attack (CVA/TIA) or completely asymptomatic who were found to have LE or CPF on transesophageal echocardiogram without other obvious causes, such as atrial fibrillation. 

From the resulting cohort from July 2018 to June 2024, we have identified a total of 10 patients who had a CVA due to a LE or CPF, or those with incidental findings of LE or CPF without a CVA/TIA. Nine patients presented with CVA/TIA, who were found to have LE or CPF on TEE without other obvious causes. One asymptomatic case with an incidental finding of CPF was identified. Patients were followed for a minimum of six months after their initial presentation of CVA/TIA. The total cohort of patients consisted of a mean age of 69.4 years, a median age of 72.5 years (range 58-79, Table 1). Seven out of 10 (70%) of our patients were women, with a mean age of 68.6 years, a median 72 years (range 58-79 years). The co-existing medical conditions our cohort had were HLD (4/10), HTN (7/10), DM (5/10), CAD (2/10), and CKD (1/10).

## Results

Case 1: TP

**Figure 1:**
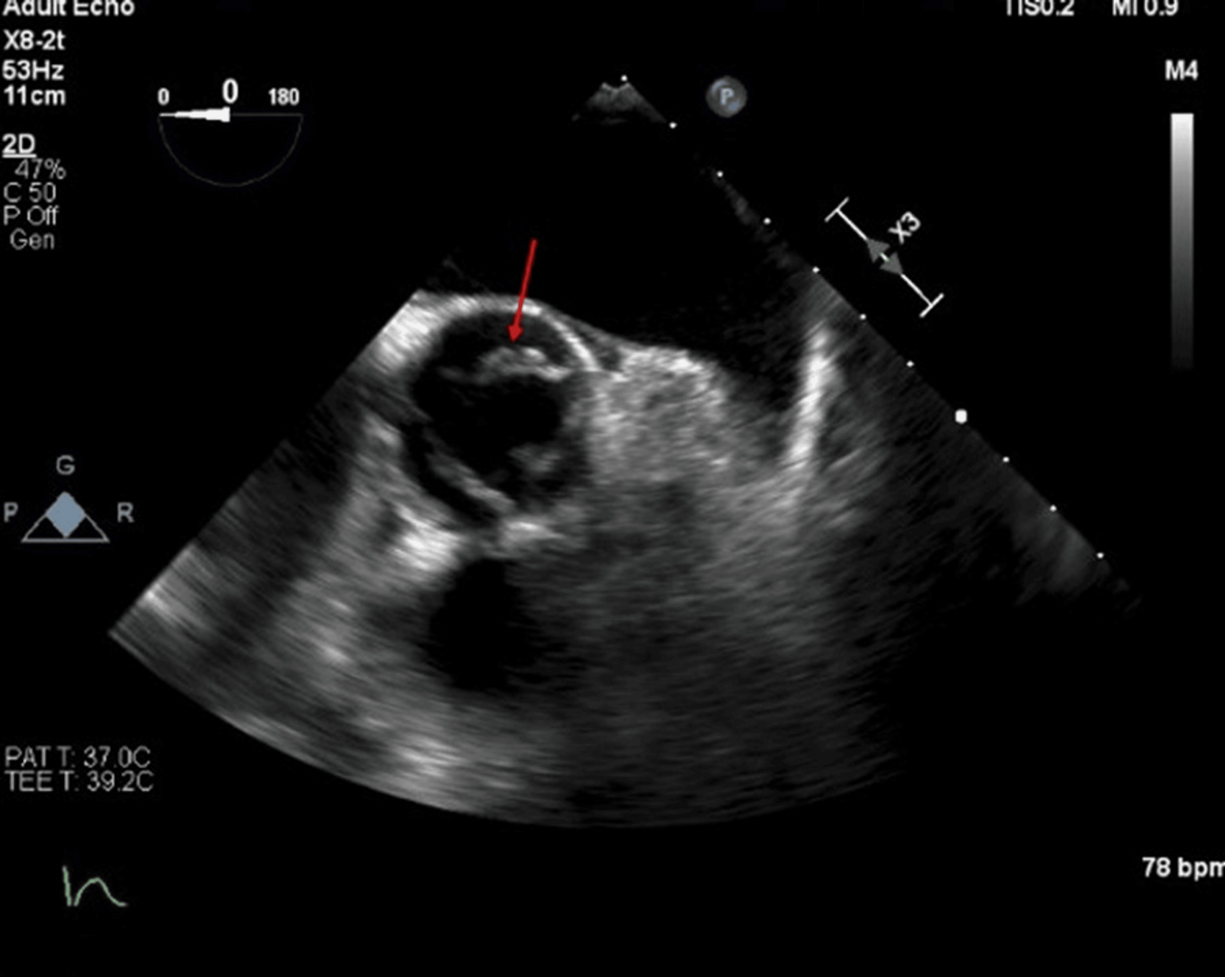
1 x 3 mm highly mobile mass on all three coronary cusps, without significant abnormalities denoted by red arrow

A left-dominant-handed 60-year-old Caucasian female patient presented with left upper arm weakness. The patient has a medical history of diabetes mellitus, hypertension, dyslipidemia, and a remote smoking history (quit in 1999). The patient was on appropriate home medication of metformin 1000mg, glimepiride 4 mg daily, atorvastatin 20 mg daily, quinapril 10 mg daily, and levothyroxine 75 mcg daily. It is unclear if the patient was compliant with medication. A physical exam revealed mild left upper extremity weakness with extremity drift as well as a slightly weak grip on the left compared to the right. No weakness was noted elsewhere or other neurological deficit. Brain CT without contrast showed no hemorrhage or acute abnormality. Neck CT angiogram (CTA) showed focal stenosis up to 50% in the proximal right internal carotid artery with no evidence of vascular occlusion, dissection, or hemodynamically significant stenosis. Bilateral carotid duplex ultrasound showed a moderate amount of plaque in the proximal internal carotid artery on the right side with 50%-69% stenosis of the vessel. Brain MRI showed a small discontinuous area of acute infarction involving the cortices of the right frontoparietal region and right temporo-occipital region. TEE showed approximately 1x3 mm highly mobile mass on all three coronary cusps, without significant abnormalities (Figure 1). These masses were consistent with LEs. Initially started on high-dose atorvastatin, aspirin 81 mg daily, clopidogrel 75 mg, but plans for 21 days of DAPT before discontinuing aspirin.

The patient was switched to anticoagulation rivaroxaban 20 mg QID and continued high-intensity statin therapy. The patient eventually underwent bioprosthetic aortic valve replacement (AVR). Post TEE showed well-placed and functional AVR. Aggressive treatment for this patient was based on her relatively young age, good baseline functional status, and clear embolic stroke pattern without other immediate causes. On six months follow-up, the patient was without recurrence of stroke and continued aspirin therapy alone. On a five-year follow-up, the patient developed a CVA due to a right carotid stenosis and underwent a right carotid endarterectomy. The patients was initiated on aspirin and clopidogrel.

Case 2: TK

A 72-year-old Caucasian male patient with a history of diabetes mellitus, coronary artery disease status post coronary artery bypass graft (CABG) x4, hypertension, and ischemic cardiomyopathy presented for a fall, transient dysarthria, and left upper extremity weakness. His wife reported the patient had slurred speech lasting for 30 minutes. In the emergency department (ED), the patient's symptoms resolved and denied any headache, visual changes, shortness of breath, chest pain, dizziness, numbness or tingling in the upper or lower extremities, or weakness. Physical examination and lab results were unremarkable. The patient was admitted for a possible transient ischemic attack (ABCD2 score of 5). Carotid/CTA neck showed no significant stenosis, dissection, or occlusion. Patient’s home medications consisted of aspirin 81 mg daily, atorvastatin 80 mg daily, fenofibrate 160 mg daily, metformin 500 mg twice daily, pioglitazone 45 mg daily, and metoprolol succinate 25 mg daily. It is unclear if the patient displayed compliance with medications. Head MRI without contrast showed acute-to-subacute infarct in the right temporal lobe and parietal lobe as well as age-related atrophy and small vessel ischemic changes. Telemetry showed a normal sinus rhythm.

**Figure 2:**
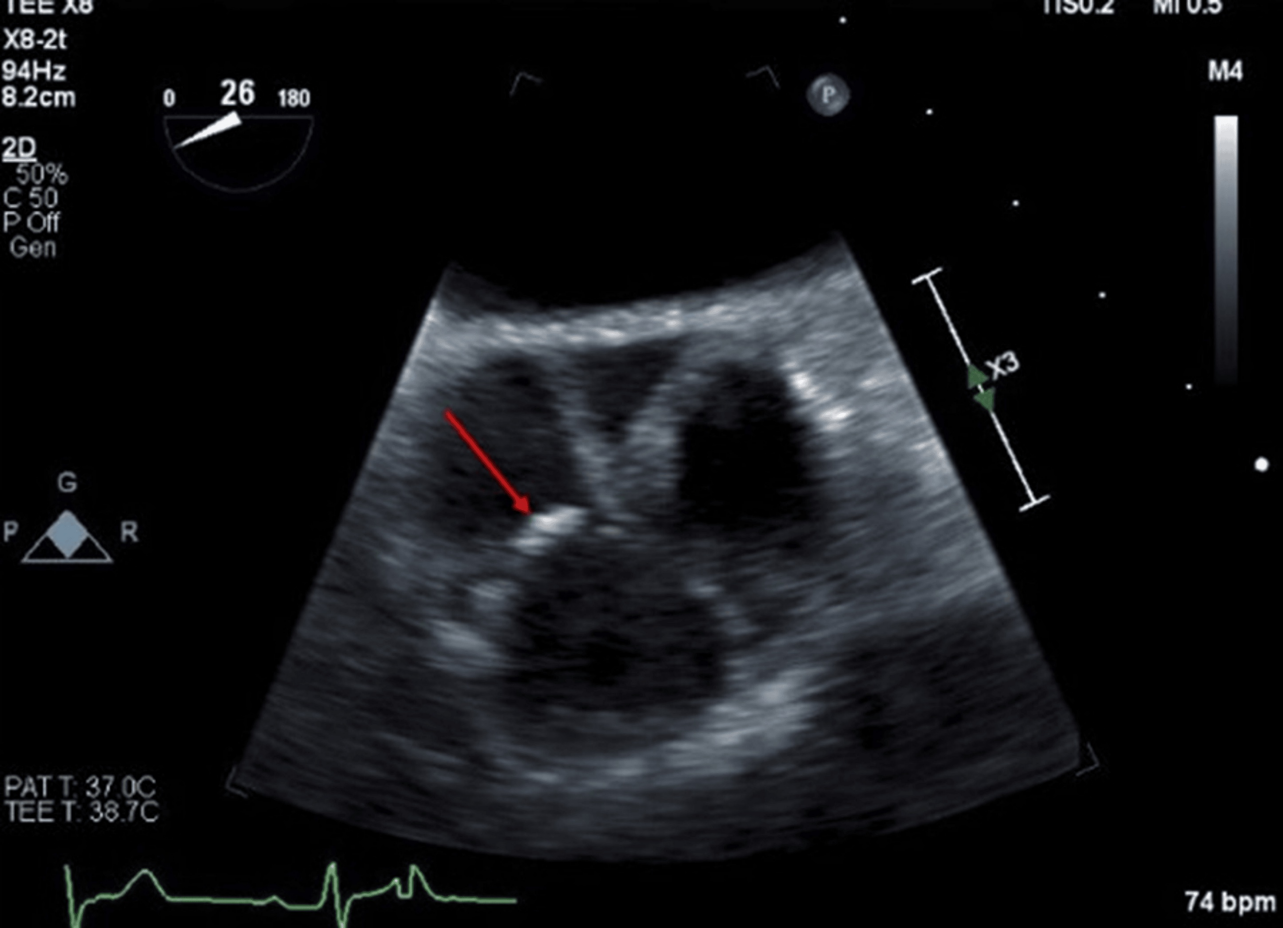
One small slender filamentous process on the tips of the non-coronary and right coronary cusps of the aortic valves measuring <1mm x2 mm long Lambl’s excrescence (red arrow)

The TEE showed one small slender filamentous process on the tips of the non-coronary and right coronary cusps of the aortic valves, measuring <1mm x2 mm long Lambl’s excrescence, suspected as the source of a cardioembolic infarct (Figure 2). The patient was treated with aspirin 81 mg and clopidogrel 75 mg daily for 21 days and thereafter with clopidogrel alone. Outpatient follow-up discussed aortic valve surgery, but no record indicated it was performed. At a 4-month follow-up, he remained stroke-free. On a four-month follow-up, the patient continued his monotherapy of clopidogrel treatment and remained stroke-free. Three years later, the patient presented with confusion. Head CT without contrast showed age-indeterminate infarct in the right periventricular parietal lobe, confirmed by a head MRI. The echocardiogram showed similar findings to the prior. Patient CTA neck revealed a significant left carotid stenosis, prompting a thrombectomy. His medication was changed to Eliquis (apixaban) 5 mg twice daily, and clopidogrel was discontinued.

Case 3: RB

**Figure 3:**
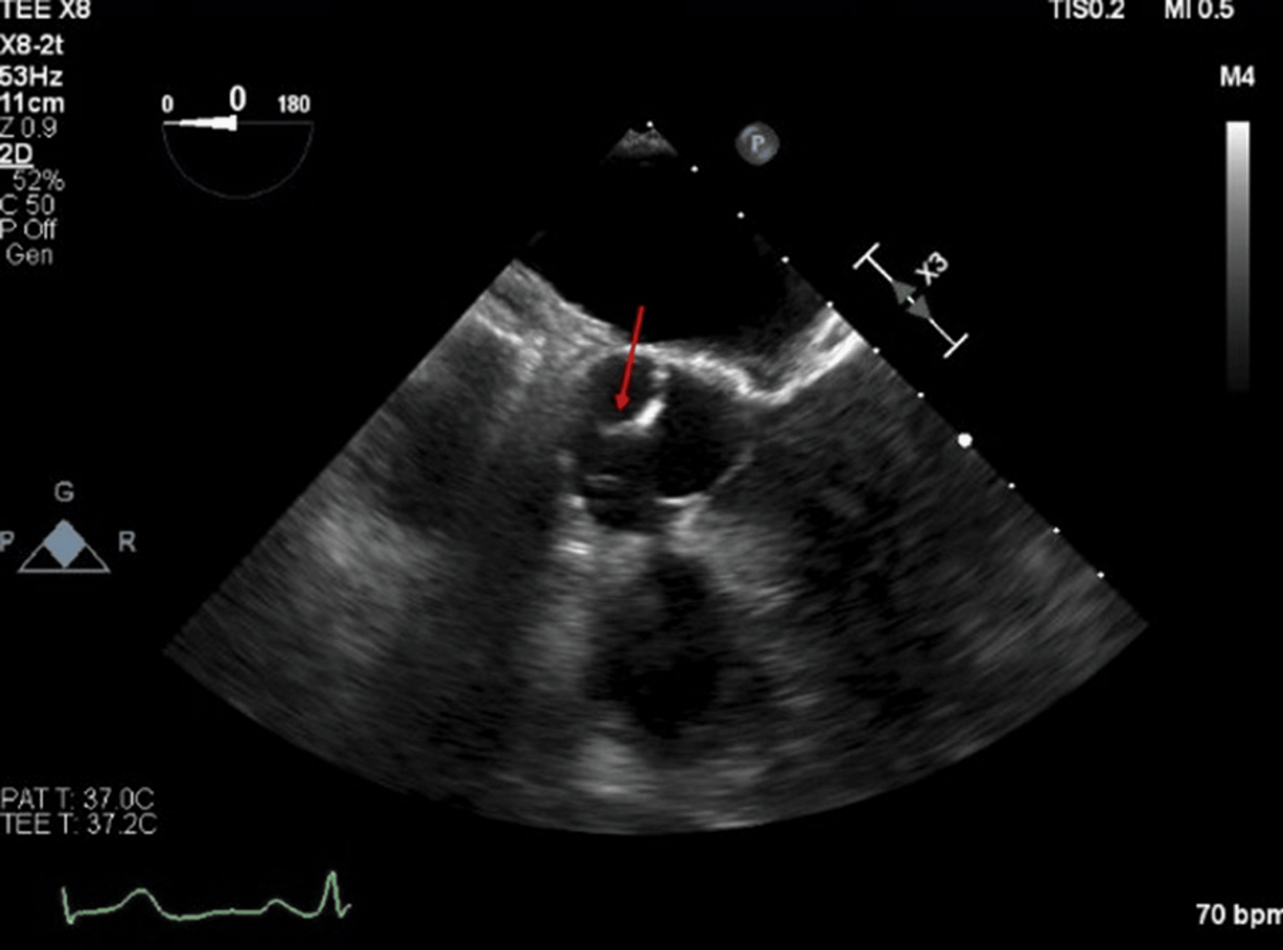
A small filamentous mobile 1 mm x 3 mm frond-like mass (red arrow) on the supravalvular non-coronary cusp of the aortic valve

A 58-year-old Caucasian female patient with a history of hypertension, hyperlipidemia, diabetes mellitus, CAD status post PCI, and hypothyroid presented with slurred speech and right arm numbness. The patient stated she had a few episodes of sudden onset of blurry vision associated with vertigo and dizziness within the past week. However, after dinner, the patient stated that she suddenly lost sensation in her right arm from the shoulder down, and her husband noticed slurred speech. The patient denied any blurry vision, double vision, headache, dizziness, chest pain, or palpitations. She is a current smoker of five to six cigarettes per day. Patient’s home medication consists of metoprolol tartrate 50 mg twice daily, insulin lispro 15 units, and insulin detemir 50 units at bedtime. It was unclear if the patient was compliant. On admission, A1c was 9% and elevated direct low-density lipoprotein (LDL) 130 mg/dL, low high-density lipoprotein (HDL) 27 mg/dL, and triglycerides 140 mg/dL. CT head was negative for acute hemorrhage or infarcts. MRI of the brain without contrast showed acute small infarcts in the bilateral cerebral hemispheres, suggestive of an embolic source. Bilateral carotid duplex ultrasound showed scattered bilateral mixed plaque and less than 50% stenosis in both carotid systems. A TEE was performed to rule out the source of the embolism, which showed a small filamentous mobile 1 mm x 3 mm frond-like mass on the supravalvular non-coronary cusp of the aortic valve (Figure 3). The echocardiogram overall showed a normal left ventricular systolic function with an ejection fraction of 65%-70%. Surgical evaluation for the removal of the mass and cardiac arrhythmia was requested. The decision to insert a Medtronic Looper recorder was made for cardiac arrhythmia, and no further surgical intervention was planned for the mass removal. The loop recorder report showed no atrial fibrillation. The patient was to continue dual antiplatelet therapy (DAPT) of aspirin 81 mg daily and Plavix (clopidogrel) 75 mg daily as well as atorvastatin 40 mg daily. On a six-month follow-up, the patient was without recurrence of stroke, and the patient was free from atrial fibrillation. On 12-month follow-up patient did not have any recurrent CVA/TIA. Two years later, the patient was found to be in new-onset atrial fibrillation and switched to apixaban.

Case 4: HV

**Figure 4:**
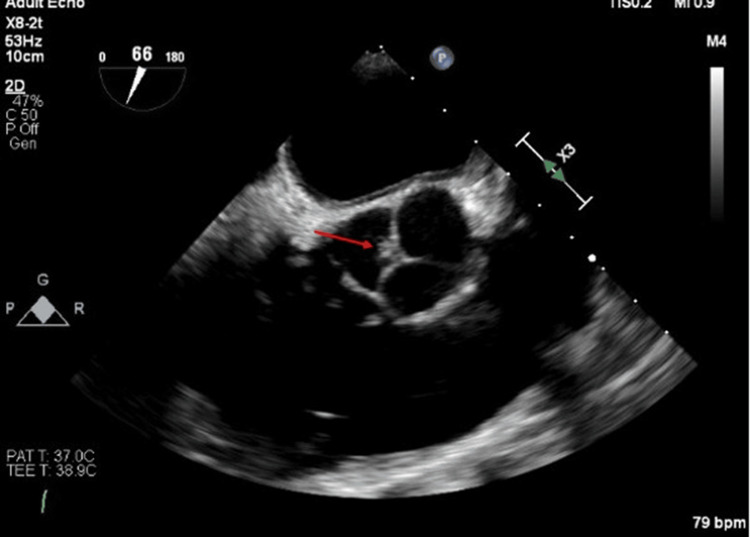
<1mm x 3mm mobile mass (red arrow) on the tip of the non-coronary cusp of the aortic valve

A 75-year-old Hispanic female patient presented to an outpatient transesophageal echocardiogram. A couple of months prior, the patient suffered from a right retinal artery occlusion (August 11, 2019). She has a medical history of chronic kidney disease stage 3 with proteinuria, resistant hypertension, and diabetes mellitus. The patient went to visit an eye emergency department regarding her vision, where there was an embolic disease of the right cilioretinal artery via history and fundus examination. Patient’s home medications consisted of losartan 50 mg daily, furosemide 40 mg daily, insulin glargine 15 units, Rosuvastatin 40 mg daily, and clonidine 0.1 mg BID. MRA showed no high-grade stenosis, and the carotid ultrasound showed very mild echoic plaque deposition producing <50% stenosis. Orbital Doppler was normal bilaterally. The patient had a lipid panel, which showed a cholesterol level 75 mg/dL, HDL level of 37 mg/dL, triglycerides level 222 mg/dL, LDL level <40 mg/dL, and non-HDL level 38 mg/dL. The patient was started on 81 mg of aspirin and continued her home dose of statin. In addition, the patient was scheduled for a two-week CardioNet and 2D echo with reflex TEE if TTE was negative. CardioNet did not show evidence of atrial fibrillation or flutter, and the patient was continued on aspirin and statin. The TEE showed <1mmx3mm mobile mass on the tip of the non-coronary cusp of the aortic valve; otherwise, left ventricular function was normal and no intracardiac shunt of the left atrium appendage was observed (Figure 4). The TEE also showed significant atherosclerosis of the descending aorta with possible intramural hematoma. CT was scheduled but was not performed because of GFR <22 mL/min/1.73m2 and CKD stage 3/4. With the TEE findings, the patient was started on rivaroxaban (Xarelto) 15 mg, and aspirin was discontinued. Eventually, due to a reduced creatinine clearance from her CKD 3, the patient’s anticoagulation was switched to Eliquis. On a 12-month follow-up, the patient did not have any recurrent stroke or TIA.

Case 5: MH

**Figure 5:**
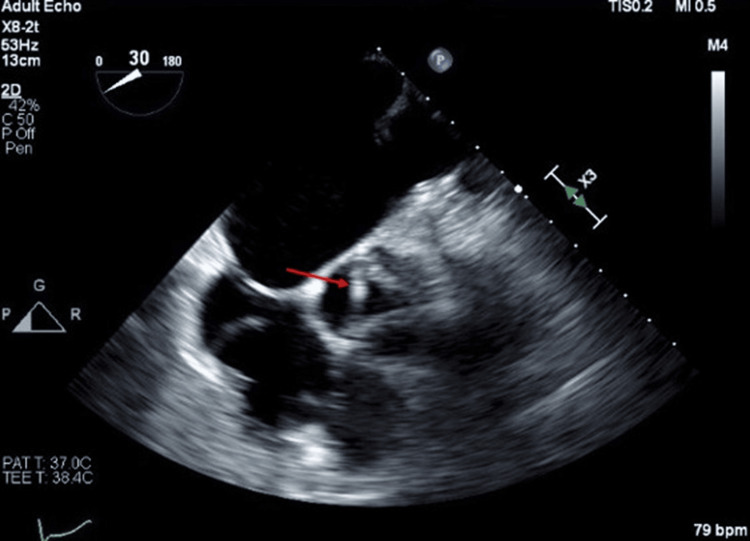
Transesophageal echocardiograph showed multiple filamentous masses on the atrioventricular (AV) leaflets compatible with fibroelastomas denoted by red arrow

A 75-year-old Caucasian female patient presented with expressive aphasia and slurred speech. The patient has a past medical history of prior CVA on full dose aspirin 325 mg, a history of atrial fibrillation status post ablation 15 years prior, and not on anticoagulation, NASH cirrhosis, hypertension, and recurrent transient ischemic attacks. The St. Jude implanted recorder showed 0.1% cumulative atrial arrhythmia burden. Patient’s home medication consisted of warfarin 5 mg daily, carvedilol 25 mg twice daily, Olmesartan 20 mg daily, Nifedipine 60 mg daily, atorvastatin 10 mg at bedtime, and glimepiride 2 mg daily. It was unclear if the patient complied with medications. The patient stated that her speech was “off” and woke up feeling well, but her symptoms of slurred speech and expressive aphasia started at 11 a.m. In the emergency department, the patient’s slurred speech resolved had generalized weakness and was negative for headache, vision changes, focal weakness, and near syncope. On physical exam, the patient presented with no neurological deficits, 5/5 bilateral upper extremity and bilateral lower extremity strength, no sensory deficits, normal finger-to-nose and rapid alternating movements, no pronator drift, steady, non-ataxic gait with normal pace and speech, no dysarthria. Head CT was negative for subacute stroke or intracranial hemorrhage. Bilateral carotid duplex ultrasound showed no hemodynamically significant stenosis. The patient could not have a head MRI due to a bladder stimulator. The TTE showed multiple filamentous masses on the atrioventricular (AV) leaflets compatible with fibroelastoma (Figure 5). A follow-up TEE showed LE noted without signs of vegetation or papillary fibroelastoma, no evidence of left atrium appendage thrombus, and no evidence of shunt with agitated saline. Given the history of CVA and TIAs as well as the new finding of LE, the patient was started on anticoagulation with warfarin 5 mg daily because the patient could not afford Eliquis. About 12 months later, the patient presented with aphasia and dysarthria for 1 hour with NIH 4 and no gross focal deficits or hemiparesis. The patient was transferred to a tertiary center for thrombectomy and discharged with apixaban 5 mg BID.

Case 6: HB

A 64-year-old female patient with a past medical history of diabetes, hypertension, hyperlipidemia, spinal stenosis, and gastroesophageal reflux disease (GERD) presents with left body weakness with slurred speech, and balance issues, accompanied by difficulty walking. She reported an earlier fall without head trauma or loss of consciousness. She denied chest pain, palpitations, shortness of breath, or visual changes. The patient is a former smoker who quit more than 30 days ago. Her family history included diabetes, hypertension, myocardial infarction (father), and multiple CVAs (brother). At home, the patient is on lisinopril 5 mg daily and insulin aspart protamine and insulin aspart (Novolog Mix) 70/30. It was unclear if the patient complied with home medication.

**Figure 6:**
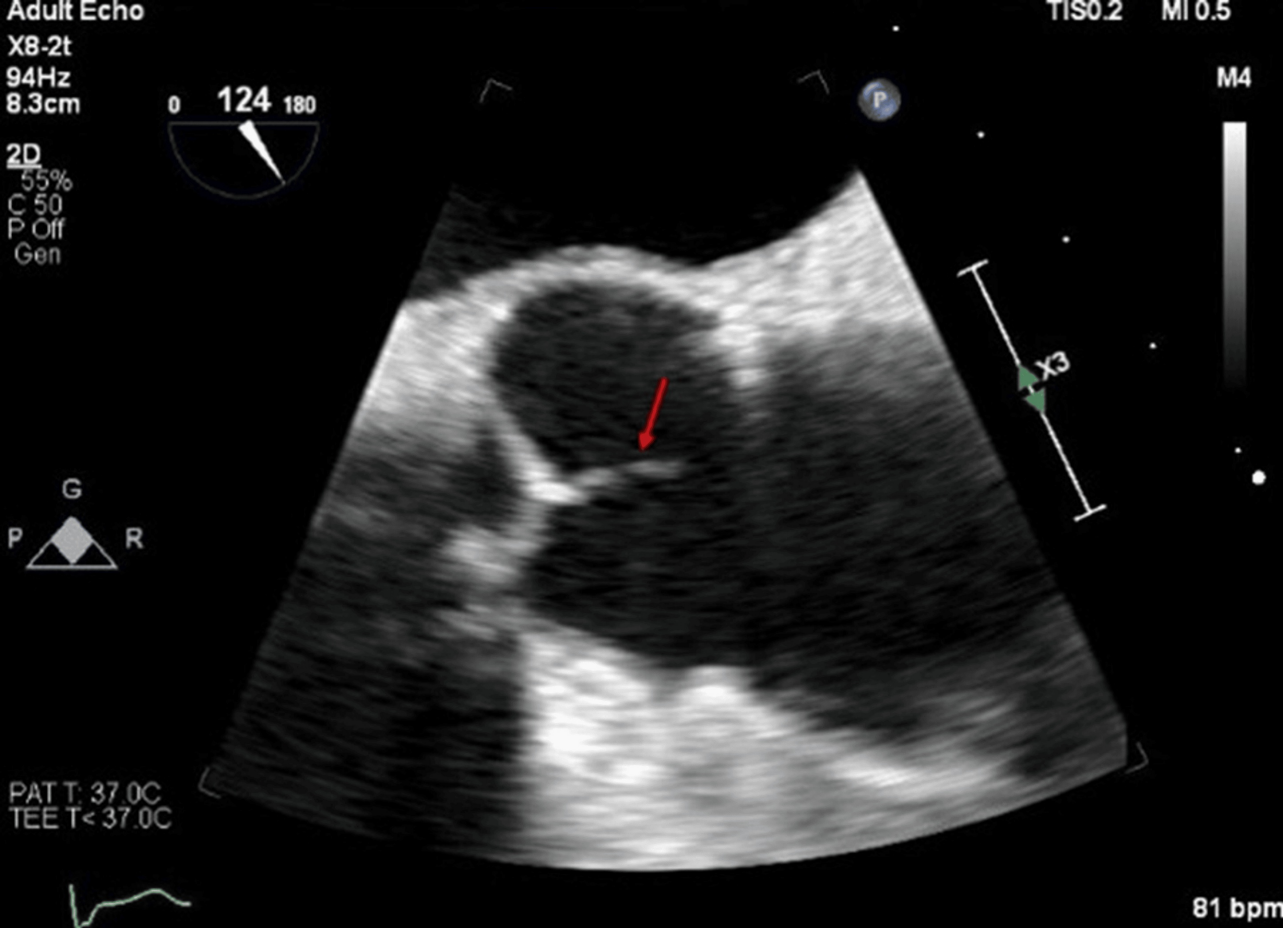
Transesophageal echocardiograph showing a small filamentous echo density visualized on the aortic side of the aortic valve, which was consistent with Lambl’s excrescence (red arrow)

On physical exam, the patient had 2/5 strength in the left upper extremity and 3/5 strength in the left lower extremity, but 5/5 strength in the right upper and lower extremities. The rest of the neurological exam was unremarkable. Brain MRI revealed acute-to-subacute infarcts in the right basal ganglia, internal capsule, and corona radiata (1x1x1.3 cm) and anterior superior right medulla (6x4x6 cm). It also showed a small chronic infarct in the inferior right cerebellar hemisphere and an abnormal increased signal in the visible distal right vertebral arterial flow-void related to occlusion or more proximal significant stenosis. The carotid ultrasound showed no significant stenosis in either internal carotid artery but indicated right vertebral artery stenosis/occlusion or hypoplasia, with antegrade flow in both vertebral arteries. The TEE showed a small filamentous echo density visualized on the aortic side of the aortic valve, which was consistent with LE; no thrombus was detected in the left atrial appendage; injection of contrast showed no interatrial shunt (Figure 6). The patient was treated with aspirin 81 mg daily and atorvastatin 40 mg daily and discharged to acute rehab. There, the patient developed deep vein thrombosis (DVT) in her left arm and was started on apixaban (Eliquis) 5 mg BID. On a 12-month and two-year follow-up, the patient did not develop any strokes or TIA.

Case 7: FL

**Figure 7:**
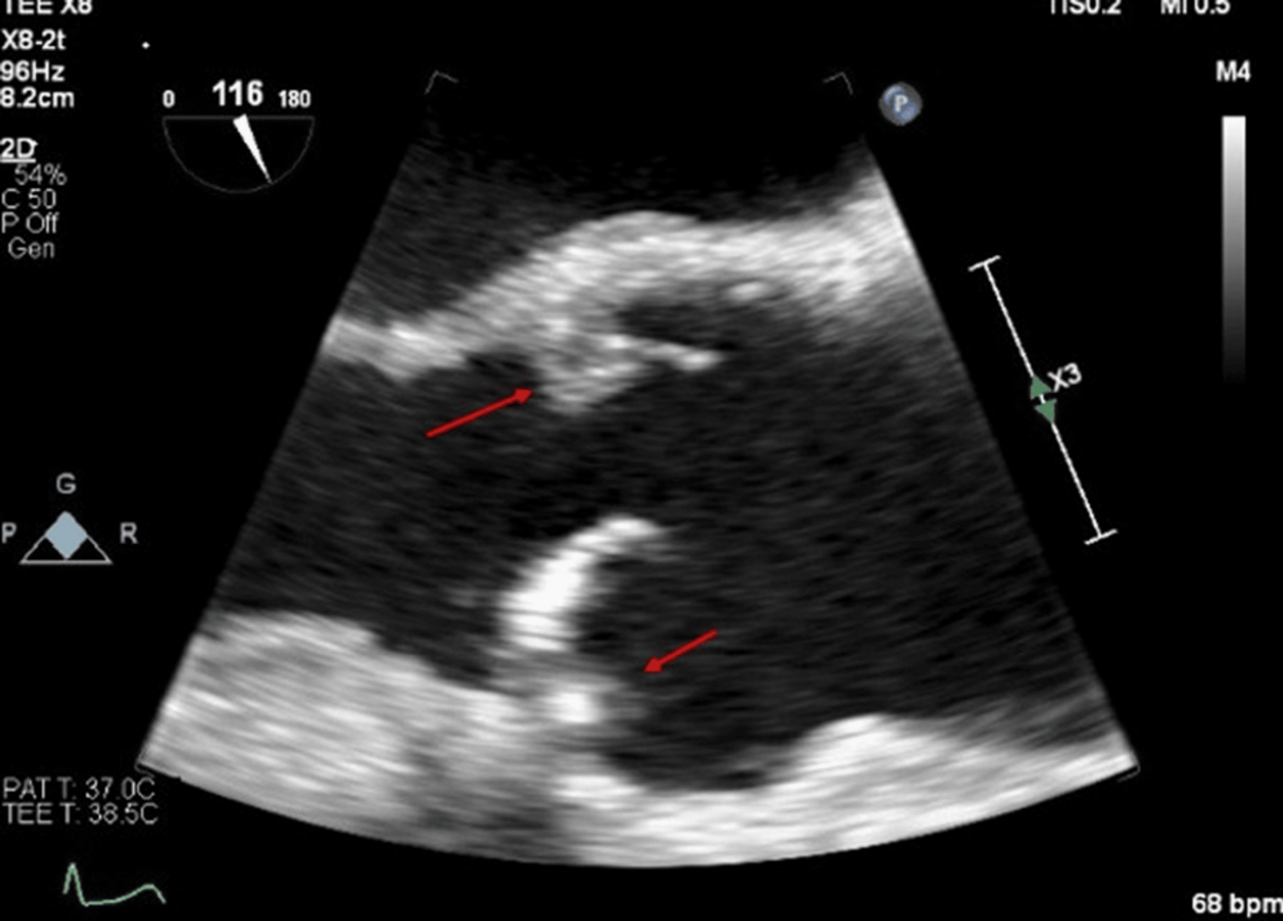
9Transesophageal echocardiograph showed fibroelastomas on atrioventricular (AV) leaflets and lipomatous hypertrophy of the interatrial septum noted on the right and noncoronary cusps with the largest measuring 0.1 cm by 1.0 cm denoted by the red arrows.

A 73-year-old male patient with a past medical history of aortic valve fibroelastoma, hypertension, and hyperlipidemia had a recent diagnosis of retinal artery embolism and presented to the office for a follow-up. Patient’s home medication consisted of amlodipine 5 mg daily, atorvastatin 20 mg daily, and hydrochlorothiazide 12.5 mg daily. Documentation did not report on compliance with home medication. The patient’s bilateral carotid duplex ultrasound showed mild-to-moderate fibrocalcific plaques of the bilateral common carotid to internal carotid arteries without hemodynamically significant stenosis. A TEE showed fibroelastomas on AV leaflets and lipomatous hypertrophy of the interatrial septum noted on the right and noncoronary cusps, with the largest measuring 0.1 cm by 1.0 cm (Figure 7). Since the retinal artery embolism and being on Xarelto, the patient had no recent hospitalization, emergency room, or urgent care visits. The patient remains a non-smoker and has been compliant with the medical regimen and dietary restrictions. In addition, the patient’s pharmacologic stress test using regadenoson showed no evidence of ischemia. Myocardial perfusion is normal without scintigraph evidence of infarction or inducible ischemia. During stress imaging, the left ventricular ejection fraction (LVEF) is 59%. On a 12-month follow-up patient remained asymptomatic without developing a stroke or TIA.

Case 8: JR

A 79-year-old female patient with migraines, PVCs, GERD, recent diagnosis of hyperthyroidism, and prior history of DVT (completed three-month course of Xarelto) presenting to the emergency department for evaluation of slurred speech. She was admitted to the hospital for acute cerebrovascular accident evaluation. Patient's home medications are metoprolol succinate 50 mg daily, Zetia 10 mg daily, and omeprazole 40 mg daily. Documentation did not report on compliance with home medication. The patient's NIH score was 1 on admission. Neck CT angiogram showed atherosclerotic plaque of the proximal carotid arteries with less than 10% stenosis. Brain MRI showed chronic lacunar infarcts in the right dominance and medial right cerebral hemisphere; acute to subacute infarct of the left basal ganglia/corona radiata measuring 12x9 mm and in the cortical/subcortical high left parietal region measuring 7x3 mm. With CTA not revealing any significant large vessel atherosclerosis and MRI revealing two areas of restricted diffusion in the left middle cerebral artery (MCA) distribution, it was recommended by neurology to consider cardioembolism and outpatient cardiac monitoring for occult atrial fibrillation. The 2D echo showed an ejection fraction (EF) of 55%-60%, mild diastolic dysfunction, moderate mitral regurgitation with no pulmonary hypertension, thrombus in the left atrial appendage (LAA), or pericardial effusion. She was discharged on 21 days of aspirin 81 mg oral daily and Plavix 75 mg oral daily with a high-dose statin.

**Figure 8:**
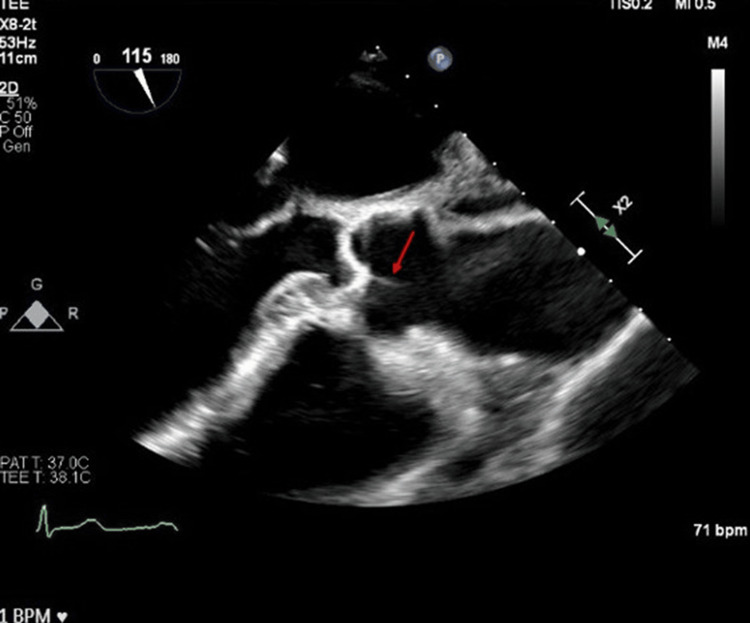
Transesophageal echocardiograph showed normal left ventricular (LV) function, trace aortic regurgitation with Lambl’s excrescence noted (red arrow), and mild mitral valve regurgitation

She followed up with her cardiologist in one month. Due to concerns about atrial fibrillation, she was scheduled for a 30-day event recorder and a TEE. The TEE showed normal left ventricular (LV) function, trace aortic regurgitation with LE noted, and mild mitral valve regurgitation (Figure 8). Patient’s 30-day event recorder showed normal sinus rhythm with frequent premature ventricular contractions (PVCs) 6% with one run of four beats of nonsustained ventricular tachycardia (NSVT) and occasional premature atrial contractions (PACs) 2%. On an eight-month follow-up, the patient did not have any recurrent CVA or TIA. With other sources being ruled out, the patient's stroke was most likely to be cryptogenic from the LE.

Case 9: CW

A 61-year-old female patient with hypothyroidism presented via virtual visit during the COVID pandemic with the sudden onset of hand numbness and difficulty with dexterity, such as weakness with grasp and finger adduction, which began 45 hours earlier after a workout. She denied headaches, chest pain, shortness of breath, or palpitations. MRI revealed small foci of restricted diffusion in the right frontoparietal region, consistent with acute or recent subacute infarcts (largest 6x4 mm). Multiple abnormalities raised suspicion of an embolic stroke, prompting further workup for an embolic source and atrial fibrillation. The patient's home med consisted of levothyroxine 88 mcg daily. The patient tested negative for COVID.

**Figure 9:**
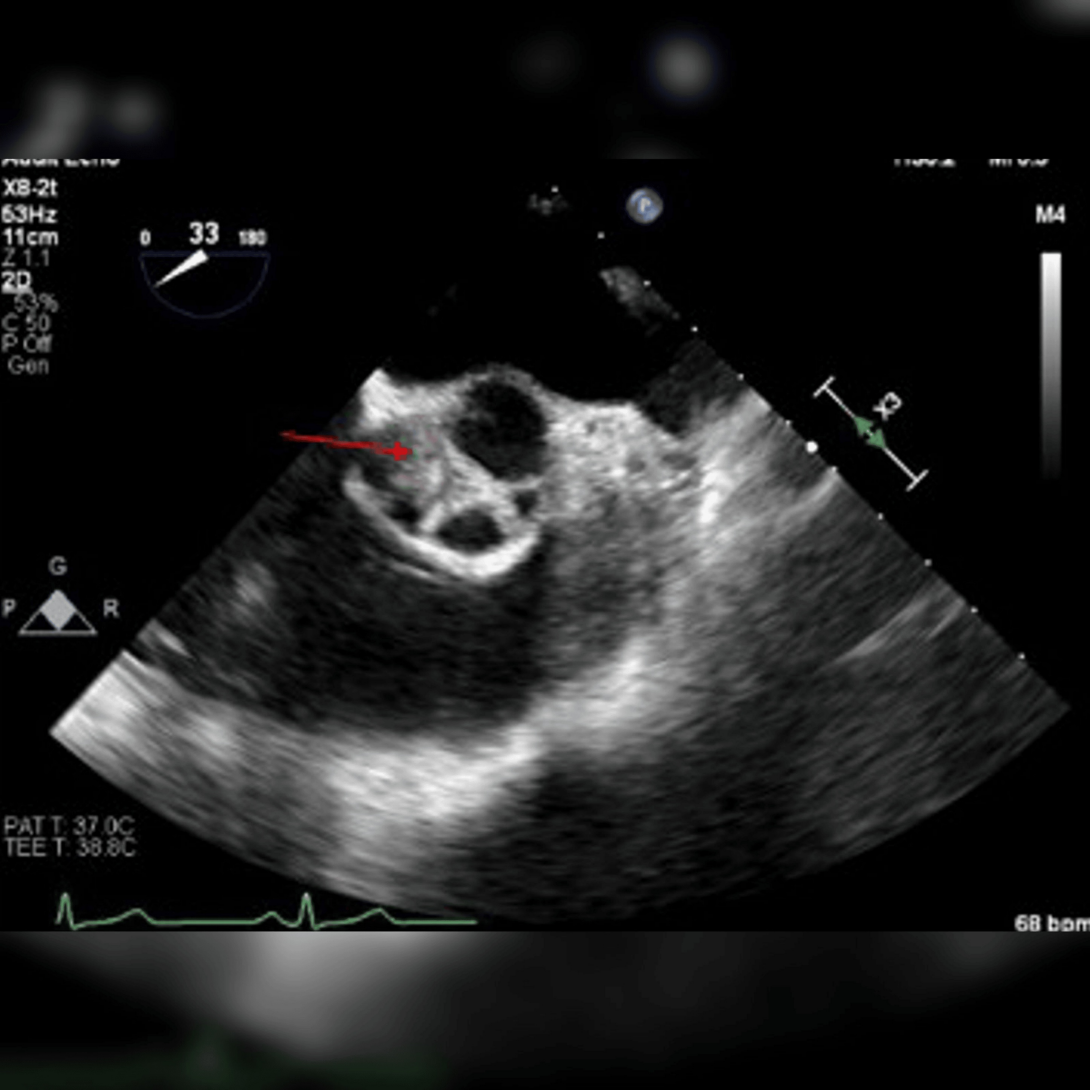
Transesophageal echocardiograph showing broad based fibroelastoma of the noncoronary cusp (red arrow)

She was started on aspirin 81 mg, clopidogrel 75 mg, and rosuvastatin 20 mg. The patient was placed on a Holter monitor, which showed the patient's rhythm was sinus rhythm without A-fib events. TTE showed an ejection fraction 65%-70%, no thrombus, and mild pulmonary hypertension. Bilateral carotid ultrasound and CT angiography of the brain and neck showed no significant stenosis or occlusion. A TEE showed several LE on the valve leaflet and a small broad-based fibroelastoma on the noncoronary cusp (Figure 9). With this new finding on the TEE, patient anticoagulation was switched from Plavix to Xarelto 20 mg daily. In a 12-month follow-up, she remained symptom-free without recurrent CVA or TIA. On a three-year follow-up, the patient’s repeat TTE showed stable findings with persistent LE on the aortic valve and fibroelastoma. A follow-up TEE 2 months later confirmed these findings of the LE on the aortic valve leaflets and small broad-based fibroelastoma on the noncoronary cusp. It appears unclear if the patient had a surgical evaluation, but on follow-up, the patient was on medical management. She remained free of recurrent CVA/TIA.

Case 10: RG

**Figure 10:**
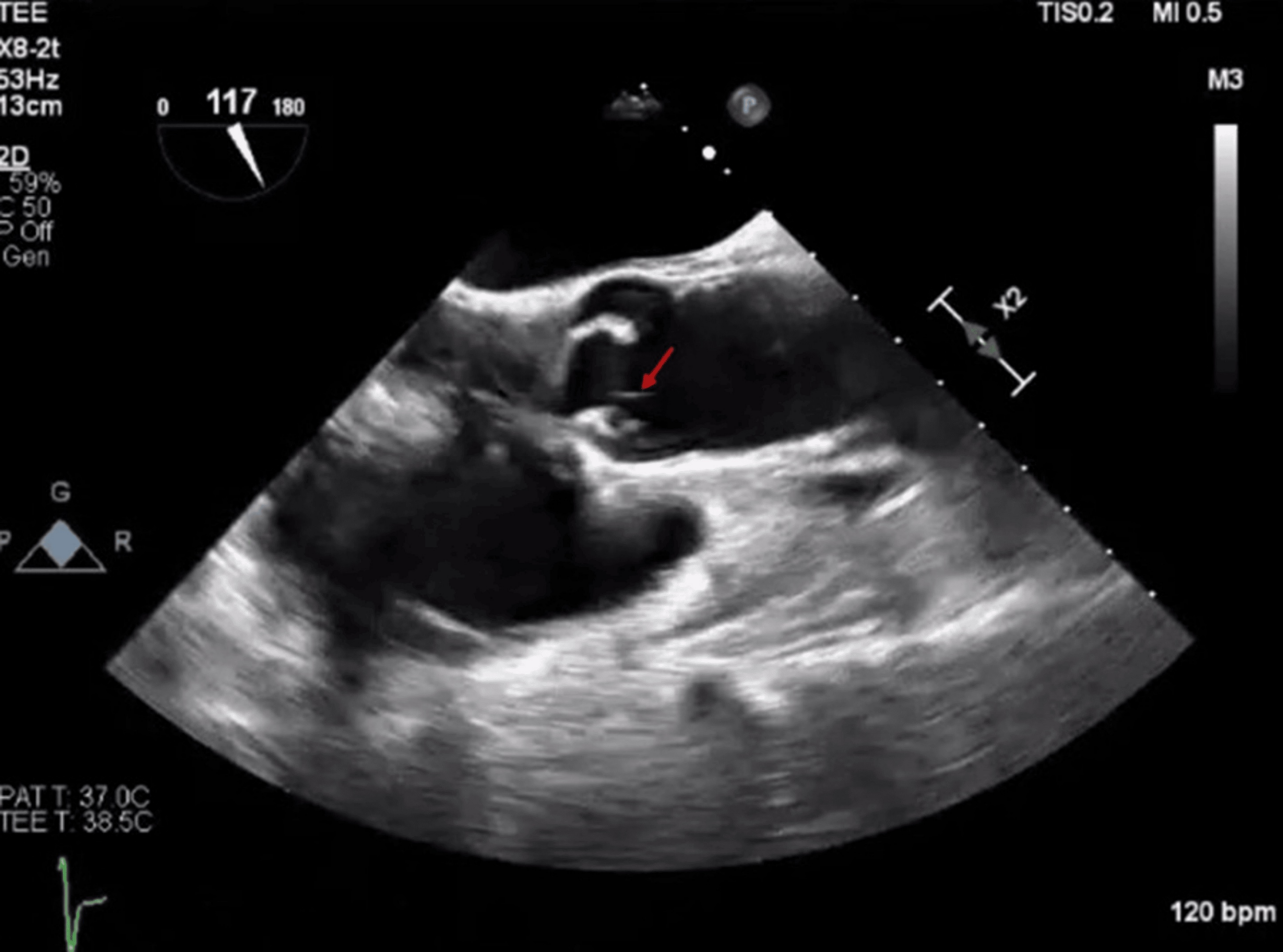
Transesophageal echocardiograph showed an ejection fraction (EF) of 50% to 55%, thrombus in the left arterial appendage, papillary fibroelastoma seen on aortic valve (red arrow), and mild mitral regurgitation

A 76-year-old male patient with chronic obstructive pulmonary disease (COPD) Type A presented with shortness of breath and was found to be in new-onset A-fib with a rapid ventricular rate. The patient’s CHA₂DS₂-VASc Score was 2. The acronym CHA₂DS₂-VASc stands for congestive heart failure, hypertension, age (≥75, doubled), diabetes, stroke (doubled), vascular disease, age (65-74), and sex (female). The patient was seen by cardiology and started on metoprolol and rivaroxaban (Xarelto). The patient required supplemental oxygen 2 L nasal cannula. At baseline, the patient did not use any oxygen. TTE showed EF 61%, normal functioning LV ejection fraction, mild mitral annular calcification, and mild tricuspid regurgitation. His TEE showed EF of 50%-55%, thrombus in the left arterial appendage, papillary fibroelastoma seen on the aortic valve, and mild mitral regurgitation (Figure 10). The patient had a cardioversion but reverted to A-fib. Thus, the patient was placed on amiodarone and metoprolol. He followed up with his cardiologist regarding this hospitalization. The patient continued his Xarelto for anticoagulation and rate and rhythm control medications for his A-fib. Regarding the papillary fibroelastoma, due to the size appearing small and not significantly mobile, it was decided to continue to monitor with an echocardiogram. A repeat echo in six months demonstrated normal LV function with aortic sclerosis, with no mention of fibroelastoma. On a 12-month follow-up from the initial findings of the papillary fibroelastoma, the patient was free of any strokes or TIA. Two years from the initial findings, the patient was free from CVA/TIA but had to be switched from rivaroxaban to apixaban due to cost.

For the patients with CVA/TIA, no other identifiable causes of cerebrovascular events were identified. Five patients received direct oral anticoagulants (rivaroxaban or apixaban), one received warfarin, three received aspirin plus P2Y12 inhibitors, and one patient underwent valvular replacement (Table 1). Two patients on follow-up had a stroke due to carotid artery stenosis about three or five years after the initial stroke from the LE and CPF. These two patients were managed with carotid endarterectomy or thrombectomy. Importantly, none of the 10 patients had recurrent hospital visits for transient ischemic attacks or ischemic strokes from the LE/CPF, except two patients who sustained a recurrent CVA from carotid artery stenosis. Interestingly, one patient in the cohort had an incidental finding of CPF and was placed on rivaroxaban and then switched to apixaban due to cost. On a two-year follow-up, patients did not have sustained recurrent CVA.

**Table 1:** A summary the each patient treatment and course DM: Diabetes mellitus, HTN: hypertension, HLD: hyperlipidemia; CAD: coronary arterial disease; CABG: coronary artery bypass graft; CKD: chronic kidney disease; CVA: cerebrovascular accident; NASH: non-alcoholic steatohepatitis; TIA: transient ischemic attack; DVT: deep vein thrombosis; COPD: chronic obstructive pulmonary disease

Patient	Age	Sex	Past Medical H istory	Charlson Comorbidity Score	CPF or LE	Initial Diagnosis	Stroke Location	Recurrent Stroke from LE or CPF	Initial treatment	Timeline*
Case 1: TP	61	F	DM, HTN, HLD	1	LE	CVA	Anterior right parietal cortex, parts of the insular region of the right temporal lobe	No	21 days of DAPT and high dose statin, then switched to rivaroxaban, aortic valve replacement, afterwards patient continued aspirin therapy alone	0 month - CVA, DAPT -> Rivaroxaban; 6 months - AVR and aspirin alone, no recurrent CVA/TIA; 5 years - patient CVA due to right carotid stenosis s/p R carotid endarterectomy, ASA and clopidogrel tx
Case 2: TK	72	M	DM,CAD s/p CABG x4, HTN,ischemic cardiomyopathy	3	LE	CVA	Right temporal lobe and a small portion of the adjacent parietal lobe	No	Aspirin and clopidogrel	0 month - CVA; 4 months - no recurrent CVA/TIA; 3 years - CVA, left carotid stenosis s/p thrombectomy switched to Apixaban
Case 3: RB	58	F	HTN, HLD, DM, CAD s/p PCI, hypothyroid	2	CPF	CVA	Bilateral cerebral hemisphere	No	Aspirin and Clopidogrel	0 month - CVA; 6 months - no recurrent CVA/TIA; 12 months - no recurrent CVA/TIA; 2 years - new onset of afib started on Apixaban
Case 4: HV	75	F	CKD 3, HTN, DM	3	LE	Retinal artery embolism	Right cilioretinal artery	No	Apixaban	0 month - retinal artery embolism; 12 months - no recurrent CVA/TIA
Case 5: MH	75	F	hx CVA, hx afib s/p ablation (15 years no AC), NASH cirrhosis, HTN, recurrent TIA	5	LE	TIA	N/A due to bladder stimulator	No	warfarin	0 month - CVA ; 12 months - CVA switched to Apixaban
Case 6: HB	64	F	DM, HTN, HLD, spinal stenosis, GERD	1	LE	CVA	Posterosuperior right basal ganglia/internal capsule, right corona radiata, superior right medulla, inferior right cerebellar hemisphere	No	Aspirin -> Apixaban in 1 month	0 month - CVA; 1 month - switched to Apixaban due to DVT at rehab; 12 months - no recurrent CVA/TIA; 2 years - no recurrent CVA/TIA
Case 7: FL	73	M	Aortic valve fibroelastoma, HTN, HLD	2	CPF	Retinal artery embolism	Retinal arterial branch occlusion	No	Rivaroxaban	0 month - retinal artery embolism; 12 months - no recurrent CVA/TIA
Case 8: JR	79	F	Migraines, PVCs, GERD, hyperthyroidism, and prior history of DVT (completed 3-month course of Xarelto)	1	LE	CVA	Left basal ganglia/corona radiata, left parietal region, restricted diffusion in left MCA	No	Aspirin and clopidogrel	0 month – CVA; 8 months - no recurrent CVA/TIA
Case 9: CW	61	F	Hypothyroidism	0	Both	CVA	Right frontoparietal region	No	Aspirin and clopidogrel -> Rivaroxaban	0 month - CVA, ASA and clopidogrel; 2 months - switch Rivaroxaban after fibroelastoma identified; 12 months - no recurrent CVA/TIA; 3 years - no recurrent CVA/TIA
Case 10: RG	76	M	COPD type A	1	CPF	Asymptomatic	N/A	No	Rivaroxaban and serial monitoring	0 month - Incidental finding; 12 months - free from CVA/TIA from initial diagnosis; 2 years - free from CVA/TIA, switched from Rivaroxaban to Apixaban due to cost
*Each timestamp is from the initial incident										

## Discussion

Cryptogenic stroke (CS) is defined as cerebral ischemia of obscure or unknown origin and approximately one-third of ischemic strokes are cryptogenic [2]. The cause of CS remains undetermined because the event is transitory or reversible. Strokes could be classified as cryptogenic after an extensive evaluation. A systematic classification arose from the TOAST (Trial of Org 10,172 in Acute Stroke Treatment) study, which classified ischemic strokes based on five potential etiologies: large artery atherosclerosis, cardioembolic, small vessels occlusion, other determined etiology or of undetermined source (i.e., cryptogenic) [3].

LE are histopathologically similar to CPF. LEs are highly mobile, filamentous growths on cardiac valves, most often the aortic and mitral, which have been implicated in cryptogenic or cardioembolic strokes. They were first described in 1856 by a Czech physician, Vilem Dusan Lambl as “filiform valvular fronds with hypermobility” [1, 4]. Whereas CPF are benign cardiac tumors that are described as larger, more gelatinous, usually found away from the line of valve closure and covered by multiple layers of endothelial cells. While LE are smaller, located at the line of valve closure, and covered by a single endothelial layer. The growth of LE may be limited due to their exposed high-stress location. In contrast, CPFs are usually found on the mechanically less pressured parts of valves and other parts of the endocardium. Thus, CPF are bulkier, can be attached to the valve through a stalk or pedestal, and their surfaces may contain multiple fingerlike projections [1].

Current diagnostic criteria for LE and papillary fibroelastoma (PFE) are not universally standardized, but both are primarily diagnosed by imaging, especially TEE. The American Society of Echocardiography states that while echocardiography - especially TEE - can strongly suggest the diagnosis of both LE and PFE, definitive diagnosis requires histopathologic confirmation, as imaging alone cannot always reliably distinguish between these entities, particularly in atypical cases [5]. Histopathology remains the gold standard, as some LEs and PFEs are microscopically indistinguishable, and separation is based on gross and clinical features rather than unique imaging findings [6,7]. Therefore, without surgical excision and histopathology, the diagnosis based on TEE is best described as the most probable diagnosis, not a secured/definite one.

Literature on the management of CPFs or LEs in patients with and without stroke is currently limited and standard guidelines regarding the management of LEs are not well established. Management of patients with CPFs or LEs is based on anecdotal case reports and is most often individualized on a case-to-case basis [8]. There are no randomized trials that explore the use of anticoagulation versus antiplatelet therapy in patients with embolic cerebral infarction resulting from LE or CPF [9]. Both pharmacological and surgical approaches have been described in the management of LEs. If the workup is negative without any identifiable cause, then the patient can be treated with an antiplatelet agent such as aspirin and clopidogrel/dipyridamole and anticoagulation with coumadin. In some cases, surgical excision may benefit.

In our small cohort, there were no recurrent strokes observed due to LE or CPF, and most patients were women. In contrast, a literature review by Kariyanna et al. reported a nearly equal prevalence of LE among men (56%) and women (44%), with a 30% recurrence rate of stroke attributed to LE [10]. Furthermore, the literature review made recommendations similar to our cohort to treat with dual antiplatelet after the first episode of CV, but majority of the management is shared decision-making between clinician and patient as well as dependent on the clinical status of the patient [10]. Studies on embolic stroke of undetermined sources (ESUS) have high risk of recurrence like stroke from LE and CPF. For ESUS, studies investigated stroke prevention with direct oral anticoagulant (DOAC) compared to stroke prevention with aspirin monotherapy. All three trials showed anticoagulation with DOAC was not superior to aspirin monotherapy in presentation of recurrent stroke in ESUS patients [11].

There was one patient in the cohort that developed a CVA during the COVID-19 pandemic. Although her event occurred during the early COVID-19 pandemic, the patient tested negative for COVID-19 and did not exhibit signs of active or recent infection. Additionally, as this presentation occurred in April 2020, before the widespread availability of COVID-19 vaccines, vaccination-related hypercoagulability was unlikely. Therefore, COVID-19 or vaccination-associated stroke was not considered a probable etiology in this case.

Furthermore, the question begets whether surgical excision is indicated for CPF or LE. Surgical excision is the preferred management for symptomatic CPF, especially in patients with a history of embolic events (e.g., TIA, stroke), and for asymptomatic patients with highly mobile or large tumors. While there is no universally accepted size threshold, tumors greater than 1 cm - particularly those exceeding 1.5 cm - are more likely to be considered for surgery, especially if mobile or valvular in location [12-17].

Surgical resection is curative, with low operative risk and excellent long-term outcomes, and valve-sparing techniques are typically feasible. Surgery is also considered if CPF is discovered incidentally during other cardiac operations [12,13,16]. For asymptomatic, small (<1 cm), and non-mobile CPFs, conservative management with close clinical and echocardiographic surveillance is reasonable, as the risk of embolization is lower in this group. In patients who are not surgical candidates due to comorbidities or patient preference, long-term anticoagulation or antiplatelet therapy may be considered, although evidence for efficacy is limited and based on observational data [14,15,17,18].

In contrast, LEs are generally managed conservatively, as their causal relationship with embolic events is unproven and they are frequently found in both healthy individuals and patients with stroke. Surgical excision is not routinely indicated for LEs, even in the setting of embolic events, unless there is recurrent embolism despite optimal medical therapy and no other identifiable source [14,15,18].

While our study focused on clinical presentation and standard imaging, we acknowledge that incorporation of advanced imaging techniques (e.g., perfusion imaging, microembolic signal detection) and emerging biomarkers could further improve risk stratification and therapeutic decision-making. These tools may help identify patients at higher embolic risk and guide the need for surgical versus medical management in future studies.

Ultimately, treatment decisions should be individualized, considering clinical presentation, lesion characteristics (size, mobility, location), comorbidities, and patient preference. Serial TEE imaging is recommended for conservative management to detect changes that might prompt surgical reconsideration.

Limitations

A key limitation of this case series study is its small sample size, with only 10 patients identified over six years. This limits the generalizability and statistical power of the findings. Additionally, the study relies on retrospective data, which may be subject to information bias or incomplete records, particularly regarding follow-up care and outcomes. Due to the study being observational, there is a lack of a standardized treatment protocol with management decision based on individual cases rather than consistent criteria. This variability complicates comparisons and conclusions about the effectiveness of different treatments, such as anticoagulation versus antiplatelet therapy. Furthermore, the absence of randomized controlled trials (RCTs) or a control group makes it difficult to establish causal relationships between treatments and outcomes. Given the rarity of LE and CPF, our single-center series of 10 patients is descriptive and not powered for hypothesis testing; the goal was to highlight heterogeneity in presentation and management rather than to establish treatment efficacy.

Additionally, the diagnosis of LE and PFE in this series relied solely on imaging, primarily transesophageal echocardiography. The American Society of Echocardiography states that, while echocardiography can strongly suggest these diagnoses, definitive distinction between LE and PFE requires histopathologic confirmation, as imaging alone cannot always reliably differentiate these entities, particularly in atypical cases. Some lesions are microscopically indistinguishable, and histopathology remains the gold standard for diagnosis [1-3]. Therefore, in the absence of surgical excision and tissue diagnosis, the findings in this series should be considered the most probable diagnosis based on imaging, rather than a definitive diagnosis.

Another limitation of the study is that patients have risk factors for stroke. Although other causes were ruled out and considered cryptogenic, it does not necessitate a routine attribution of cryptogenic stroke to Lambl’s stroke. The current consensus in the medical literature is that Lambl’s excrescences are frequently detected by TEE in both healthy individuals and patients with stroke, and their causal relationship to embolic events remains unproven and controversial. Large prospective studies have found no significant association between Lambl’s excrescences and cerebrovascular disease, and they are not considered established cardioembolic substrates [19].

Lastly, another limitation of this case series is that, because only in-hospital information was available and primary care records could not be accessed, it was not possible to systematically assess medication compliance for comorbidities. The American College of Cardiology emphasizes that accurate assessment of medication adherence and comorbidity control is essential for understanding risk and optimizing management in patients with multimorbidity and recommends structured tools such as the Morisky Medication Adherence Scale for this purpose; however, these require data beyond what is typically available during hospitalization alone. While the Charlson Comorbidity Index was calculated for each patient using available hospital data, with only three out of 10 patients having a score ≥3, this approach may underestimate the true burden of comorbid disease and does not capture the adequacy of chronic disease management or adherence to prescribed therapies. The inability to access outpatient records for compliance with medications or pharmacy refill data limits the ability to fully evaluate the control of comorbidities and medication adherence, and this should be acknowledged as a limitation in the analysis [20-22].

## Conclusions

Given the many treatment options for patients with LE or CPF, we cannot infer a superior option. Treatment options should be made in conjunction with the patient’s preferences and other medical conditions considered. Patients with low bleeding risks were prescribed anticoagulation. In patients with higher bleeding risk, standard-of-care TIA treatment with DAPT was employed. This strategy appeared safe in our small cohort. The optimal treatment for CVA related to LE and CPF continues to be unknown. Large-scale randomized controlled trials are needed.

## Appendices

**Table 2:** International Classification of Diseases, 10th revision (ICD-10) Code for Lambl’s excrescences (LE) and cardiac papillary fibroelastomas (CPF).

Diagnosis	ICD-10 Code
Accident cerebrovascular	163.9
Aortic valve fibrosis	135.8
Aortic disease	135.9
Aortic valve vegetation	133.0
Benign neoplasm of heart	D15.1
